# Supplementary material for: Reverse mutational scanning of SARS-CoV-2 spike BA.2.86 identifies epitopes contributing to immune escape from polyclonal sera
Source: Nat Commun. 2025 Jan 18;16:809. doi: 10.1038/s41467-025-55871-5 (PMC11743207; doi:10.1038/s41467-025-55871-5)
Supplement: Supplementary file 3 — Description of Additional Supplementary Files [file 41467_2025_55871_MOESM3_ESM.pdf]

Description of Additional Supplementary Files

File Name: Supplementary Data 1
Description: Contains information on the participants' vaccination histories, age, and sex.

File Name: Supplementary Data 2
Description: Contains neutralization values for sera against the assayed pseudo-viruses in reference to each participant plasma sample.

File Name: Supplementary Data 3
Description: Contains information on cell lines, catalogue numbers, and primers.
