# Supplementary material for: Reverse mutational scanning of SARS-CoV-2 spike BA.2.86 identifies epitopes contributing to immune escape from polyclonal sera
Source: Nat Commun. 2025 Jan 18;16:809. doi: 10.1038/s41467-025-55871-5 (PMC11743207; doi:10.1038/s41467-025-55871-5)
Supplement: Supplementary file 7 — Reporting Summary [file 41467_2025_55871_MOESM7_ESM.pdf]

nature portfolio

Corresponding author(s): Luka Cicin-Sain

Last updated by author(s): Aug 2, 2024

Reporting Summary

Nature Portfolio wishes to improve the reproducibility of the work that we publish. This form provides structure for consistency and transparency in reporting. For further information on Nature Portfolio policies, see our Editorial Policies and the Editorial Policy Checklist.

Statistics

For all statistical analyses, confirm that the following items are present in the figure legend, table legend, main text, or Methods section.

n/a	Confirmed	
☐	☒	The exact sample size (*n*) for each experimental group/condition, given as a discrete number and unit of measurement
☐	☒	A statement on whether measurements were taken from distinct samples or whether the same sample was measured repeatedly
☐	☒	The statistical test(s) used AND whether they are one- or two-sided<br>*Only common tests should be described solely by name; describe more complex techniques in the Methods section.*
☒	☐	A description of all covariates tested
☐	☒	A description of any assumptions or corrections, such as tests of normality and adjustment for multiple comparisons
☐	☒	A full description of the statistical parameters including central tendency (e.g. means) or other basic estimates (e.g. regression coefficient) AND variation (e.g. standard deviation) or associated estimates of uncertainty (e.g. confidence intervals)
☐	☒	For null hypothesis testing, the test statistic (e.g. *F*, *t*, *r*) with confidence intervals, effect sizes, degrees of freedom and *P* value noted<br>*Give P values as exact values whenever suitable.*
☒	☐	For Bayesian analysis, information on the choice of priors and Markov chain Monte Carlo settings
☒	☐	For hierarchical and complex designs, identification of the appropriate level for tests and full reporting of outcomes
☒	☐	Estimates of effect sizes (e.g. Cohen's *d*, Pearson's *r*), indicating how they were calculated

Our web collection on statistics for biologists contains articles on many of the points above.

Software and code

Policy information about availability of computer code

Data collection	IncuCyte GUI software (versions 2019B Rev1 and 2021B) for counting eGFP positive cells
Data analysis	Graphpad prism version 10.0.0 - Microsoft office Excel 16- Biorender- Pymol 2.5.5- AlphaFold2

For manuscripts utilizing custom algorithms or software that are central to the research but not yet described in published literature, software must be made available to editors and reviewers. We strongly encourage code deposition in a community repository (e.g. GitHub). See the Nature Portfolio guidelines for submitting code & software for further information.

Data

Policy information about availability of data

All manuscripts must include a data availability statement. This statement should provide the following information, where applicable:

- Accession codes, unique identifiers, or web links for publicly available datasets
- A description of any restrictions on data availability
- For clinical datasets or third party data, please ensure that the statement adheres to our policy

Values for neutralization titers and information on plasma donors are provided in Supplementary Files 1 and 2. Data underlying the generation of the figures are provided as a source data file: Source Data.

Research involving human participants, their data, or biological material

Policy information about studies with human participants or human data. See also policy information about sex, gender (identity/presentation), and sexual orientation and race, ethnicity and racism.

Reporting on sex and gender	The sex of the participants was disclosed in this study (briefly within the materials and methods section and in a more detailed manner within supplementary file 1, gender was not disclosed or described as it does not bare relevance to our study. The assignation of sex was based on self reporting. All donors provided written consent for the plasma donation and use for research purposes. Participants also provided consent for the publication of identifiers such as sex and age.
Reporting on race, ethnicity, or other socially relevant groupings	No reporting on race, ethnicity, or socially relevant groupings in reported in our data as it does not bare relevance to the nature of our study.
Population characteristics	A brief description of population characteristics (including age, sex, last documented SARS-CoV-2 infection, number of SARAS-CoV2 boosters, and documented dates for booster administrations) was described in the materials and methods section. Additionally, a more detailed breakdown of all this information is provided within supplementary file 1.
Recruitment	All participants are part of the COVID-19 contact study, to monitor anti-SARS-CoV-2 immune responses in healthcare workers at the Hannover Medical School (MHH).Selection was done randomly, based of willingness to volunteer. No personal biases were involved in the selection of the study participants.
Ethics oversight	The collection of all plasma samples was approved by the research ethics committee of the Institutional Review Board of Hannover Medical School (8973 BO K 2020). All donors provided written consent for the blood donation and use for research purposes.

Note that full information on the approval of the study protocol must also be provided in the manuscript.

Field-specific reporting

Please select the one below that is the best fit for your research. If you are not sure, read the appropriate sections before making your selection.

☒ Life sciences ☐ Behavioural & social sciences ☐ Ecological, evolutionary & environmental sciences

For a reference copy of the document with all sections, see nature.com/documents/nr-reporting-summary-flat.pdf

Life sciences study design

All studies must disclose on these points even when the disclosure is negative.

Sample size	The total sample size of this study was based on the availability of the studied number of samples and no specific statistical test was to determine if this sample size is sufficient. The sample size is disclosed within the methods section, as well as Supplementary file 2 and across figures. For each data set, data distribution was tested by Shapiro-Wilks. A paired non-parametric two sided Friedman test was performed for non-normally distributed data. P values less than 0.05 were considered significant ns, $p > 0.05$; *, $p \leq 0.05$; **, $p \leq 0.01$; ***, $p \leq 0.001$
Data exclusions	Every pseudovirus was tested on at least 20 random plasma samples. These were arranged so that around 80% of the plasma originated from the same set of participants. This approach aimed to minimize variability due to differing inherent potencies among plasma samples. All available PVNT50 values are presented within the figure labels and source data. No data points from assayed plasma were excluded during the analysis.
Replication	Neutralization assays conducted for pseudoviruses with participant plasma were conducted with at least 20 biological replicates (i.e atleast 20 individual plasma samples were used), hence each group has at least 20 successful PVNT50 biological replicate values. Assays were conducted in technical duplicates. For duplicate measurements where a documented technical error was observed in one (false input virus number or cellular detachment) unicates were considered.
Randomization	Due to technical limitations (the limited volume of plasma samples and the technical challenges associated with assaying 30 samples against ~40 different pseudo-viruses in duplicates), every pseudo-virus was tested on at least 20 individual chosen plasma samples. These were arranged so that over 80% of the plasma originated from the same set of health care workers. This approach aimed to minimize variability due to differing inherent potencies among plasma samples. All data are presented as numerical titers for each study participant in the supplementary file 2. This is described within the manuscript.
Blinding	Blinding was not relevant to the study.

Reporting for specific materials, systems and methods

We require information from authors about some types of materials, experimental systems and methods used in many studies. Here, indicate whether each material, system or method listed is relevant to your study. If you are not sure if a list item applies to your research, read the appropriate section before selecting a response.

Materials & experimental systems

n/a	Involved in the study	
☐	☒	Antibodies
☐	☒	Eukaryotic cell lines
☒	☐	Palaeontology and archaeology
☒	☐	Animals and other organisms
☒	☐	Clinical data
☒	☐	Dual use research of concern
☒	☐	Plants

Methods

n/a	Involved in the study	
☒	☐	ChIP-seq
☒	☐	Flow cytometry
☒	☐	MRI-based neuroimaging

Antibodies

Antibodies used	The study is based on plasma antibodies from participant healthcare workers. No monoclonal antibodies were used. Two-fold dilutions of PLASMA samples in DMEM [1% Penicillin-Streptomycin, 1% L-Glutamine, 5% FBS] ranging from 1:100 to 1:51200 were used for our assays.
Validation	Neutralizing activity was assessed by comparison to the neutralization of the wild-type (Wuhan) pseudo-virus. All values are shown in reference to naturally occurring BA.2 or BA.2.86 variants.

Eukaryotic cell lines

Policy information about cell lines and Sex and Gender in Research

Cell line source(s)	293T were obtained from DSMZ Cat# ACC-635; RRID: CVCL_0063<br>VeroE6 were obtained from ATCC Cat# CRL-1586
Authentication	The VeroE6 cells and 293T cells were inspected visually for correct morphology.
Mycoplasma contamination	Tested and negative.
Commonly misidentified lines (See ICLAC register)	no commonly misidentified cells have been used in this study.

Plants

Seed stocks	N/A
Novel plant genotypes	N/A
Authentication	N/A
